# Changes in brain white matter structure are associated with urine proteins in urologic chronic pelvic pain syndrome (UCPPS): A MAPP Network study

**DOI:** 10.1371/journal.pone.0206807

**Published:** 2018-12-05

**Authors:** Davis C. Woodworth, Adelle Dagher, Adam Curatolo, Monisha Sachdev, Cody Ashe-McNalley, Bruce D. Naliboff, Jennifer S. Labus, J. Richard Landis, Jason J. Kutch, Emeran A. Mayer, Richard S. Lee, Marsha A. Moses, Benjamin M. Ellingson, the MAPP Research Network

**Affiliations:** 1 Department of Radiological Science, David Geffen School of Medicine, University of California-Los Angeles, Los Angeles, CA, United States of America; 2 Department of Biomedical Physics, David Geffen School of Medicine, University of California-Los Angeles, Los Angeles, CA, United States of America; 3 G. Oppenheimer Center for the Neurobiology of Stress and Resilience, David Geffen School of Medicine, University of California-Los Angeles, Los Angeles, CA, United States of America; 4 Vascular Biology Program, Boston Children’s Hospital, Boston, MA; 5 Vatche and Tamar Manoukian Division of Digestive Diseases, David Geffen School of Medicine, University of California-Los Angeles, Los Angeles, CA, United States of America; 6 Department of Psychiatry and Biobehavioral Sciences, David Geffen School of Medicine, University of California-Los Angeles, Los Angeles, CA, United States of America; 7 Department of Biostatistics and Epidemiology, Perelman School of Medicine at the University of Pennsylvania, Philadelphia, PA, United States of America; 8 Division of Biokinesiology and Physical Therapy, University of Southern California, Los Angeles, CA, United States of America; 9 Department of Urology, Boston Children’s Hospital, Boston, MA; 10 Department of Surgery, Harvard Medical School, Boston, MA; 11 Department of Surgery, Boston Children’s Hospital, Boston, MA; 12 Department of Bioengineering, David Geffen School of Medicine, University of California-Los Angeles, Los Angeles, CA, United States of America; University of Oklahoma Health Sciences Center, UNITED STATES

## Abstract

The Multidisciplinary Approach to the Study of Chronic Pelvic Pain (MAPP) Research Network has yielded neuroimaging and urinary biomarker findings that highlight unique alterations in brain structure and in urinary proteins related to tissue remodeling and vascular structure in patients with Urological Chronic Pelvic Pain Syndrome (UCPPS). We hypothesized that localized changes in diffusion tensor imaging (DTI) measurements might be associated with corresponding changes in urinary protein levels in UCPPS. To test this hypothesis, we created statistical parameter maps depicting the linear correlation between DTI measurements (fractional anisotropy (FA) and apparent diffusion coefficient (ADC)) and urinary protein quantification (MMP2, MMP9, NGAL, MMP9/NGAL complex, and VEGF) in 30 UCPPS patients from the MAPP Research Network, after accounting for clinical covariates. Results identified a brainstem region that showed a strong correlation between both ADC (R^2^ = 0.49, P<0.0001) and FA (R^2^ = 0.39, P = 0.0002) with urinary MMP9 levels as well as a correlation between both ADC (R^2^ = 0.42, P = 0.0001) and FA (R^2^ = 0.29, P = 0.0020) and urinary MMP9/NGAL complex. Results also identified significant correlations between FA and urinary MMP9 in white matter adjacent to sensorimotor regions (R^2^ = 0.30, P = 0.002; R^2^ = 0.36, P = 0.0005, respectively), as well as a correlation in similar sensorimotor regions when examining ADC and urinary MMP2 levels (R^2^ = 0.42, P<0.0001) as well as FA and urinary MMP9/NGAL complex (R^2^ = 0.33, P = 0.0008). A large, diffuse cluster of white matter was identified as having a strong correlation between both ADC (R^2^ = 0.35, P = 0.0006) and FA (R^2^ = 0.43, P<0.0001) with urinary NGAL levels. In contrast, no significant association between DTI measurements and VEGF was observed. Results suggest that elevated MMP9 or MMP9/NGAL in UCPPS may be related to degenerative neuronal changes in brainstem nuclei through excitotoxicity, while also facilitating synaptic plasticity in sensorimotor regions.

## Introduction

In 2007, the National Institute of Diabetes and Digestive and Kidney Diseases (NIDDK) began using the classification of Urological Chronic Pelvic Pain Syndrome (UCPPS) to group a variety of chronic pain syndromes into a single classification [1]. Chronic pain syndromes now classified under the term UCPPS are Chronic Prostatitis/Chronic Pelvic Pain Syndrome (CP/CPPS) and Interstitial Cystitis/Painful Bladder Syndrome (IC/PBS). Two main factors were important in the development of this new classification: largely unsatisfactory patient response to treatment for pain, and unknown etiology [2, 3]. In response to the need for a better understanding of the pathophysiology of UCPPS, and with the hope of finding new avenues for successful treatments for the condition, the Multidisciplinary Approach to the Study of Chronic Pelvic Pain (MAPP) Research Network was created. The MAPP Research Network was established to address the lack of understanding of the etiology and pathophysiology of UCPPS by focusing on several key areas: epidemiology of the disease, phenotyping of urological symptoms, phenotyping of non-urological symptoms, neuroimaging and neurobiology of UCPPS, discovery and validation of peripheral (blood and urinary) biomarkers, and development of translational animal models of UCPPS [4, 5].

The MAPP Research Network has identified several alterations in brain structure and function using functional MRI [6–8], volumetric anatomic imaging [9, 10], and diffusion tensor imaging (DTI) [11–13]. Converging evidence from these studies and others [14] have identified unique functional, structural, and microstructural changes within regions of the sensorimotor network, and cingulate cortical areas that correlate with pain and symptom severity scores. Concurrent with these efforts, the MAPP Research Network has identified six novel non-invasive urinary proteins as biomarker candidates for UCPPS relating to inflammatory, tissue remodeling and/or vascular processes [15], including vascular endothelial growth factor (VEGF) and its receptor VEGFR1, matrix metalloproteinase-2 (MMP2), MMP9, neutrophil gelatinase-associated lipocalin (NGAL, Lipocalin 2), and the MMP9/NGAL complex. Similar to neuroimaging biomarker candidates, several of these urinary proteins were also found to be strongly associated with pain and urinary severity. In a study by Dagher *et al* this association was observed for MMP9, MMP9/NGAL and VEGFR1 in males and for all of the proteins in females [15].

Independent investigations have identified an apparent association between inflammatory or vascular biomarkers and DTI changes suggesting a possible biological link between the observed urinary protein levels and microstructural brain changes in syndromes like UCPPS. Studies have shown that MMPs play a role in angiogenesis and inflammation [16–18], as well as being involved in tissue remodeling [19, 20] and altering blood-brain barrier permeability [21], the latter of which has been shown to alter diffusion MRI measurements in the brain [22–24]. In fact, studies in stroke victims [25–27] have identified a strong association between MMP9 and diffusion MR measures of the apparent diffusion coefficient (ADC), an association which was confirmed by gene knockdown of MMP9 activity in mouse studies [28]. Additionally, MMP9 has been implicated in structural plasticity of dendritic spines [29], suggesting the possibility that increased expression of MMP9 due to peripheral inflammatory processes may concurrently facilitate increased brain plasticity in UCPPS.

In the current study, we explored possible neuroanatomical associations between urinary biomarker levels and DTI measurements in patients with UCPPS. We hypothesized that the reported protein biomarker levels are positively correlated with ADC, and negatively correlated with fractional anisotropy (FA), suggesting a neurodegenerative process that may be facilitated by elevated inflammation and its related consequences including tissue remodeling and neovascularization. Additionally, we hypothesize that these correlations will be strongest in spatially localized regions of the brain responsible for pain modulation and central control of micturition, including the raphe nuclei and the locus coeruleus complex.

## Materials and methods

### Patient population

All subjects provided informed written consent to participate in the current study. All consenting procedures and protocols were approved by the institutional review board at each of the participating sites, which included the University of California, Los Angeles (UCLA), and Northwestern University (NU). Detailed inclusion and exclusion criteria for UCPPS subjects for the MAPP study are outlined in Landis *et al* [5]. All urinary biomarker analyses for all proteins analyzed in this study were conducted at Boston Children’s Hospital. For the current evaluation of urine protein biomarker levels, stratified random sampling was used to select a subset of UCPPS subjects with more severe symptomatology. For a more detailed description of subject selection for urine protein biomarker analysis, see Dagher *et al* [15].

For the current investigation, a subset of UCPPS subjects with both urinary protein biomarker measurements and high-quality DTI data were included. This cohort consisted of 30 subjects, 17 males and 13 females, an average age of 40 years old (± 13 years standard deviation), and average symptom duration of 11 years (± 4 years standard deviation). Symptom severity was adjusted for by incorporating two primary symptom constructs of pain and urinary severity [30], which are based on the GUPI pain and urinary subscores and items of the ICSI. Average pain severity was 13.6 (± 5.4 standard deviation) and average symptom severity was 11.2 (± 6.5 standard deviation). **Table 1** displays demographic information and average urine biomarker concentrations for the patient cohort.

**Table 1:** UCPPS patient characteristics. Values reported as mean ± standard deviation.

N = 30	Age	Symptom Duration (yrs)	Pain Sev.	Urinary Severity	MMP9 (ng/mL)	NGAL (ng/mL)	MMP2 (ng/mL)	MMP9/ NGAL (ng/mL)	VEGF (pg/mL)
Mean	39.9	10.7	13.6	11.1	0.63	6.80	0.17	0.13	121.30
SD	13.5	12.5	5.4	6.5	1.07	6.38	0.17	0.23	180.63

### Urine protein biomarker acquisition and analysis

Briefly, urine samples were collected at study baseline and at the 6-month and 12-month follow-up visits as part of the Trans-MAPP Epidemiology/Phenotyping study [5] according to protocols established by the MAPP Biomarker Working Group and analyzed at the centralized MAPP Tissue Analysis and Technology Core. Clean-catch midstream urine was collected using alcohol-free Triad Medical-Benzalkonium chloride antiseptic towelettes (Allegro Medical, Mesa, AZ). After collection, urine was immediately frozen at -80°C and shipped to the MAPP Network Tissue Analysis and Technology Core for central processing, where it was then divided into 3mL aliquots and stored at -80°C as previously reported [31].

Urine samples were thawed, aliquoted and assayed. Total protein concentration was assessed using the Bradford method, and monospecific enzyme-linked immunosorbent assays (ELISAs; Quantikine; R&D Systems, Inc., Minneapolis, MN) were performed in duplicate in a double blinded manner for MMP2, MMP9, NGAL, MMP9/NGAL complex, and VEGF proteins according to manufacturer’s instructions. The average protein quantitation across duplicates for each protein biomarker was used in subsequent analyses. A more complete description of urine biomarker acquisition and analysis is available in Dagher *et al* [15]. Urine protein measurements were selected as to match the acquisition date as closely as possible, with the upper limit threshold set at one month between DTI acquisition and urine collection. The mean time between DTI acquisition and urine measurements was 4 days (± 5 days standard deviation), with the maximum being 14 days.

### Diffusion tensor imaging

The current study used data from two sites within the MAPP Network, Northwestern University (NWU) and the University of California Los Angeles (UCLA), as both used the same scanner model (Siemens Trio 3T) and presented high-quality DTI scans with comparable acquisition parameters including 8 *b* = 0 s/mm^2^ images, diffusion weighted images acquired in 60 or 61 directions, a *b*-value of 1000 s/mm^2^, echo time/repetition time (TE/TR) = 88ms/9500ms, slice thickness = 2mm with no gap, field of view (FOV) = 256mm, and an acquisition matrix = 128x128 (2mm isotropic voxels). (For a more thorough description of the neuroimaging acquisition methodology in the multisite MAPP study refer to Alger *et al*. [32] and previous MAPP DTI studies [11–13].

### Data Analysis and Statistical Parameter Mapping (SPMs)

Diffusion MRI scans were corrected for eddy currents and motion using the eddy correct functionality of the FSL Diffusion Toolbox (FDT) as part of FSL (FMRIB; Oxford, UK) [33]. FA and ADC were calculated using the *MRtrix* package (Brain Research Institute, Melbourne, Australia, http://www.brain.org.au/software/), and registered to the ICBM-DTI 1mm FA atlas using linear (12 direction via FSL FLIRT) and then nonlinear (via FSL FNIRT) registration on the FA images, the transforms of which were then applied to the ADC images.

The voxelwise associations were evaluated within a white matter mask (atlas thresholded at FA > 0.3), including subcortical gray matter structures such as the basal ganglia and the thalamus, as outlined previously [12, 34]. Statistical parametric maps (SPM) were generated using a general linear model (GLM) involving biomarker measurements along with covariates including age, sex, and symptom duration. Two additional covariates were selected as representative measures of the two most important factors (pain severity and urinary severity) as determined by analysis of psychometric data from the MAPP study by Griffith *et al* [35]; similar measures previously demonstrated the strongest relationship with mean DTI metrics in significantly different regions between UCPPS and HC subjects [12]. The GLM was implemented using the *3dttest++* command (https://afni.nimh.nih.gov/pub/dist/doc/program_help/3dttest++.html) from the AFNI software package (Analysis of Functional NeuroImages; https://afni.nimh.nih.gov/). *3dttest++* solves for a linear regression at each voxel, which yields slopes and respective T-statistics for the significance of the association between the voxelwise measure (ADC or FA) and the variable of interest when accounting for the other covariates. The resulting SPMs were thresholded at the voxelwise level, with significance set at *P* < 0.05 for each covariate of interest. Clusters of significant voxels, defined as contiguous voxels with *P* < 0.05, that exceeded a size threshold greater than 700 μL (equivalent to 700 voxels) where then used for further analysis. This cluster size threshold is highly conservative (approx. 3-4x larger) compared to previous studies [12, 34], which were based on permutation calculations suggested by Bullmore *et al*. [36]. For visualization, the voxelwise slope of the regression line of the covariate of interest was displayed on the significant clusters. Average diffusion MR values within significant clusters on SPMs were plotted against average urinary biomarker concentrations using GraphPad Prism (Version 7.0c; GraphPad Software, Inc.; La Jolla, CA 92037), and linear regression results were plotted including the best-fit line, 95% confidence intervals, and the R^2^ with respective *P*-value.

## Results

### Urinary MMP2 concentration and cerebral DTI measurements

**Fig 1:**
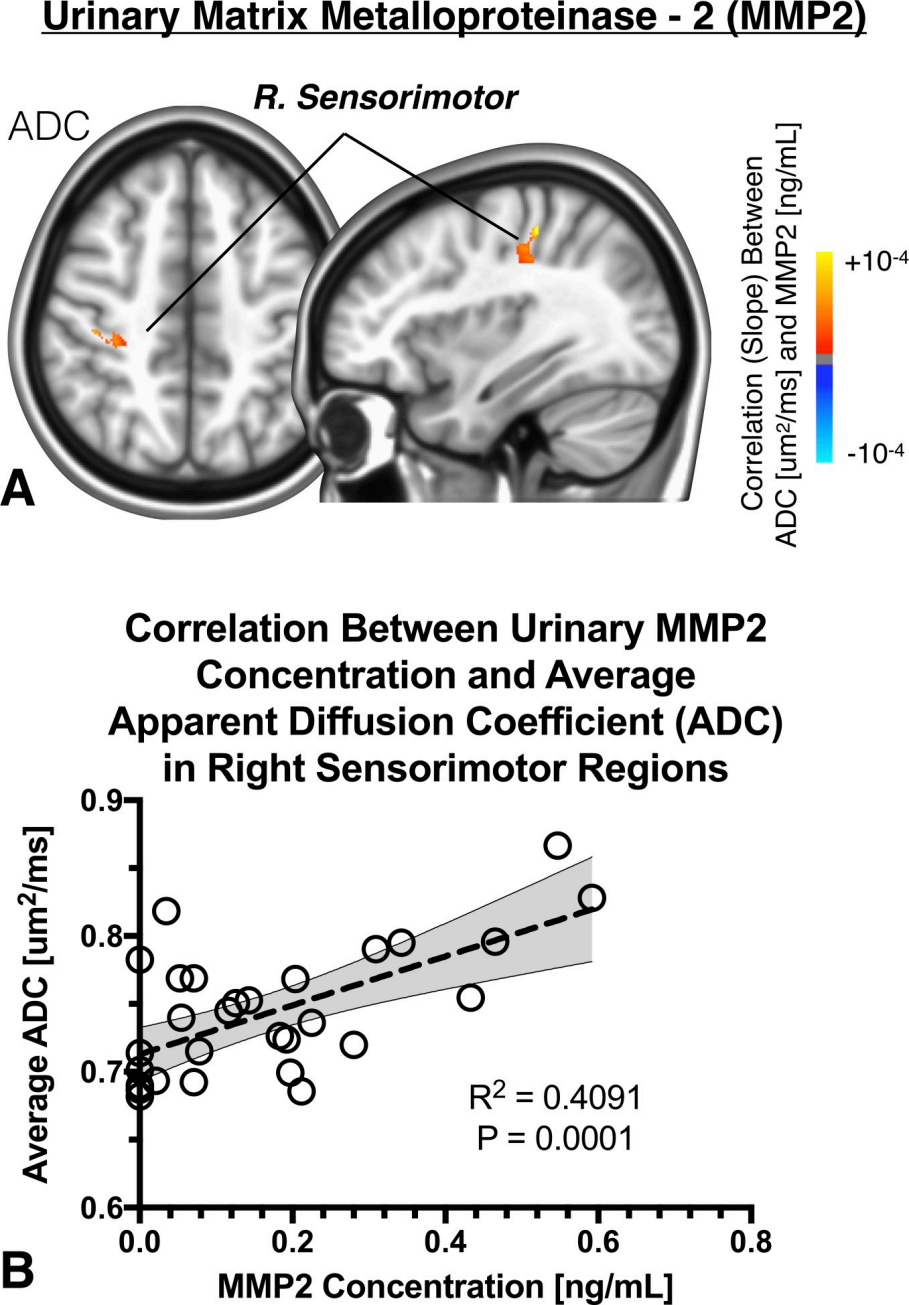
A) Anatomic localization of regions in the brain exhibiting a significant linear correlation between the apparent diffusion coefficient (ADC) and urinary protein concentration of matrix metalloproteinase-2 (MMP2) within the right somatosensory cortex. B) Linear correlation between average ADC within this cluster and urinary MMP2 concentration.

A small cluster (1.07mL) within white matter adjacent to the right motor cortex demonstrated a positive, significant correlation between urinary MMP2 concentration and ADC after accounting for age, sex, symptom duration, BPI severity and ICPI total score (**Fig 1A**). Quantification of the correlation between average ADC within this cluster and urinary MMP2 concentration showed a strong and significant association (**Fig 1B;** *Pearson’s Correlation*, *R*^*2*^ *= 0*.*4091*, *P = 0*.*0001*). No spatially-specific associations were observed between FA and MMP2 concentration on SPMs.

### Urinary MMP9 concentration and cerebral DTI measurements

**Fig 2:**
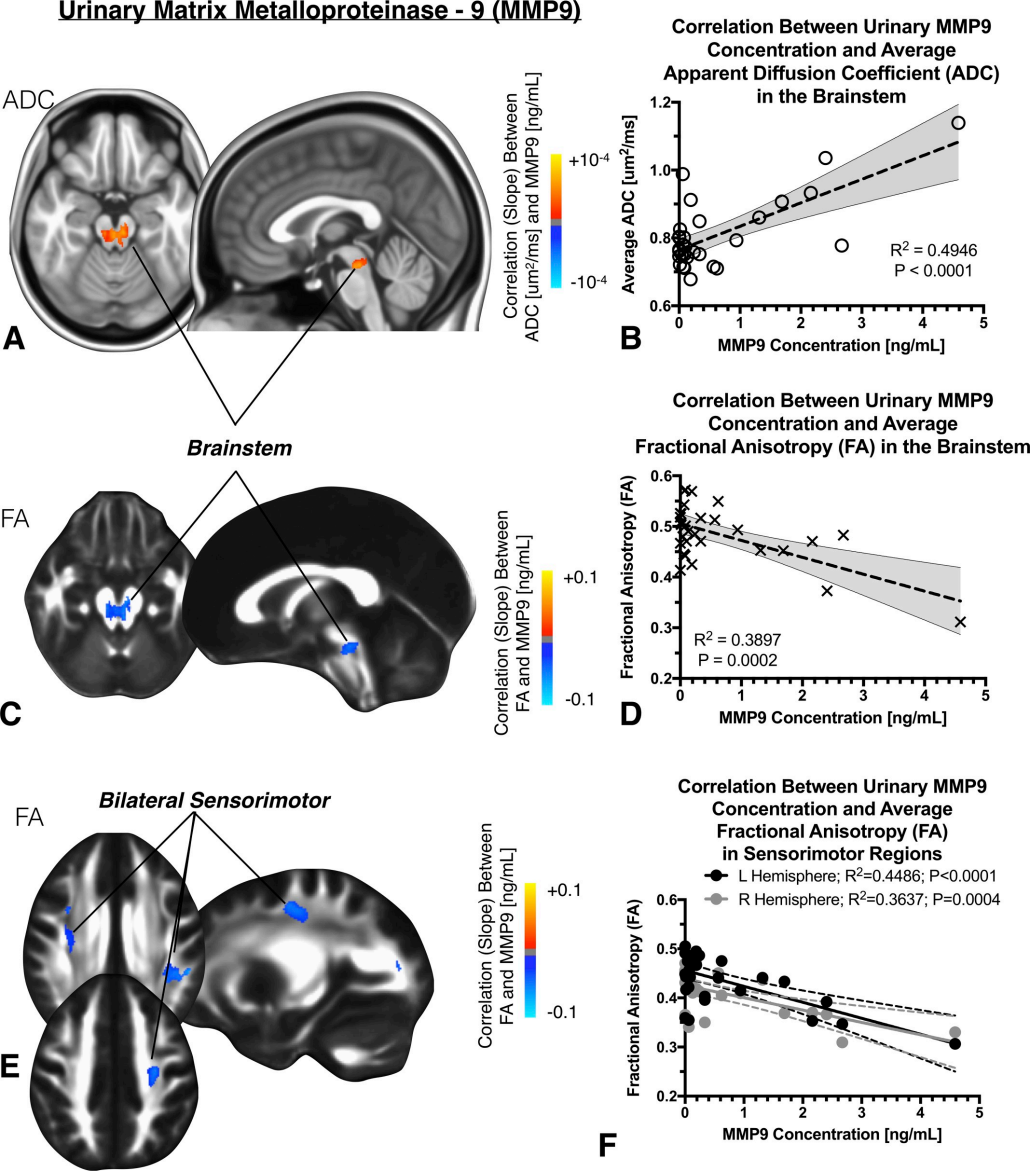
A) Anatomic localization of regions in the brain exhibiting a significant linear correlation between ADC and urinary protein concentration of matrix metalloproteinase-9 (MMP9) within the midbrain portion of the brainstem, encompassing the dorsal Raphe nuclei (DRN). B) Linear correlation between average ADC within this cluster and urinary MMP9 concentration. C) Anatomic localization of regions in the brain exhibiting a significant linear correlation between fractional anisotropy (FA) and MMP9 also present in the brainstem. D) Linear correlation between average FA within this cluster and urinary MMP9 concentration. E) A second set of clusters with a significant linear correlation between FA and MMP were localized to the sensorimotor regions, bilaterally. F) Linear correlation between average FA within these two clusters and urinary MMP9 concentration.

Two distinct regions of interest within the brain were identified as having a strong association between DTI measurements and urinary MMP9 concentration (**Fig 2**), namely a region within the brainstem and white matter connecting the sensorimotor regions, bilaterally. A 959uL cluster within the brainstem, encompassing the dorsal Raphe nuclei (DRN) and the locus coeruleus complex (locus coeruleus and Barrington’s nucleus; LCC), was identified as having a strong association between MMP9 and ADC after accounting for covariates (**Fig 2A and 2B;** *R*^*2*^ *= 0*.*4946*, *P<0*.*0001*). Similarly, a 1,005uL cluster within the midbrain was found to have a strong association between MMP9 concentration and FA, after accounting for covariates (**Fig 2C and 2D;** *R*^*2*^ *= 0*.*3897; P = 0*.*0002*). The second set of clusters were identified as having a strong correlation between MMP9 and FA within generalized sensorimotor regions, bilaterally (**Fig 2E and 2F;** *Left hemisphere*, *volume = 1*,*554uL*, *R*^*2*^ *= 0*.*4486*, *P<0*.*0001; Right hemisphere*, *volume = 961uL*, *R*^*2*^ *= 0*.*3637*, *P = 0*.*0004*). No significant difference in the slope between MMP9 concentration and FA between left and right sensorimotor regions were observed (*P = 0*.*4314*).

### Urinary NGAL concentration and cerebral DTI measurements

**Fig 3:**
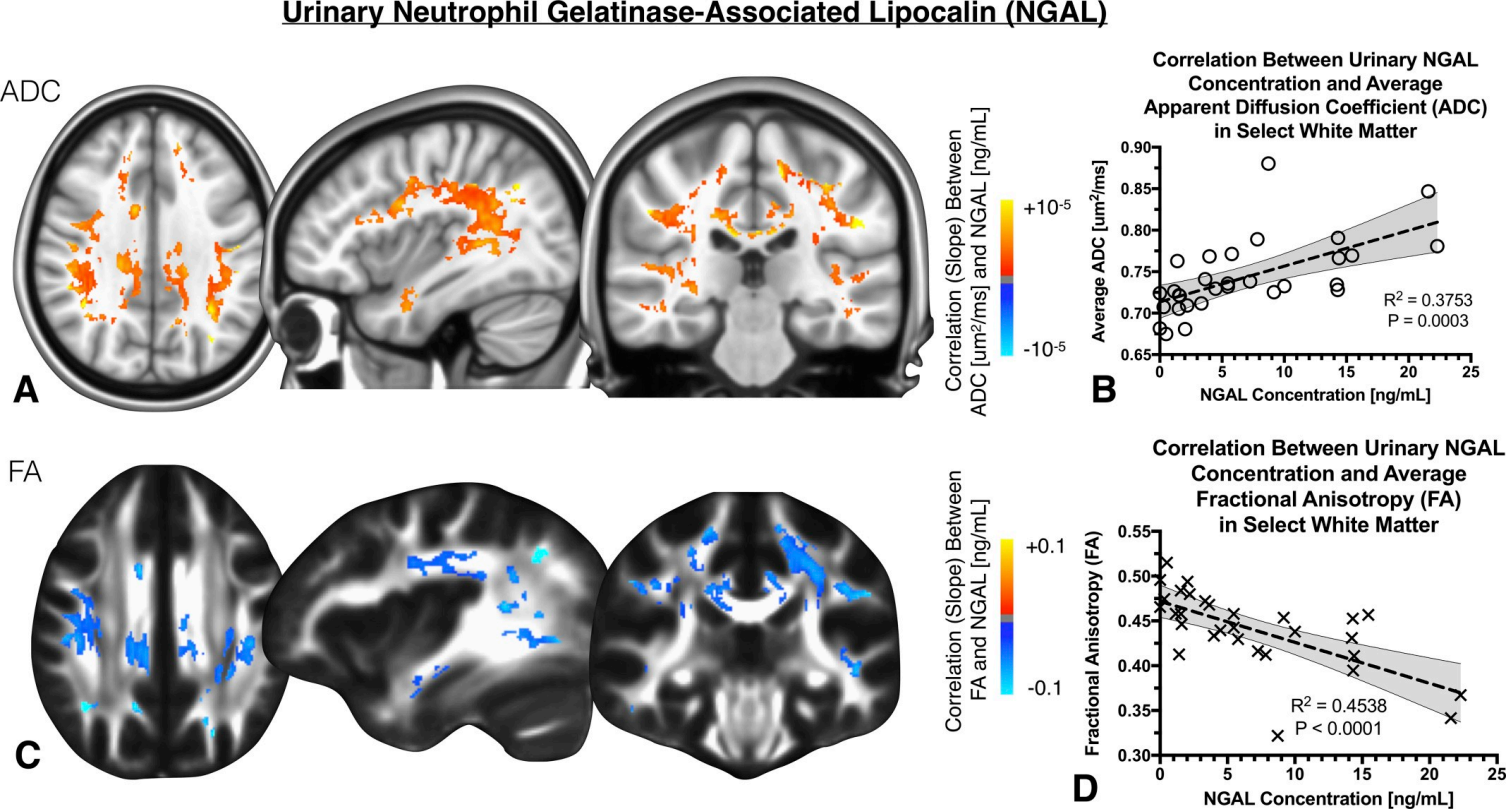
A) Anatomic localization of regions in the brain exhibiting a significant linear correlation between ADC and neutrophil gelatinase-associated lipocalin (NGAL), diffuse throughout the brain white matter. B) Linear correlation between average ADC within this cluster and urinary NGAL concentration. C) Anatomic localization of regions in the brain exhibiting a significant linear correlation between FA and NGAL, diffusely distributed in white matter. D) Linear correlation between average FA within this cluster and urinary NGAL concentration.

Diffuse regions of white matter illustrated a strong association between DTI measurements and measures of urinary NGAL concentration (**Fig 3**). Specifically, a large (46.0mL) cluster distributed throughout cerebral white matter regions showed a strong positive correlation between ADC and NGAL (**Fig 3A and 3B;** *R*^*2*^ *= 0*.*3753*, *P = 0*.*0003*). Additionally, a large (31.0mL) cluster exhibited a strong negative correlation between FA and NGAL (**Fig 3C and 3D;** *R*^*2*^ *= 0*.*4538*, *P<0*.*0001*) throughout similar regions of the brain.

### Urinary concentration of MMP9/NGAL complex and cerebral DTI measurements

**Fig 4:**
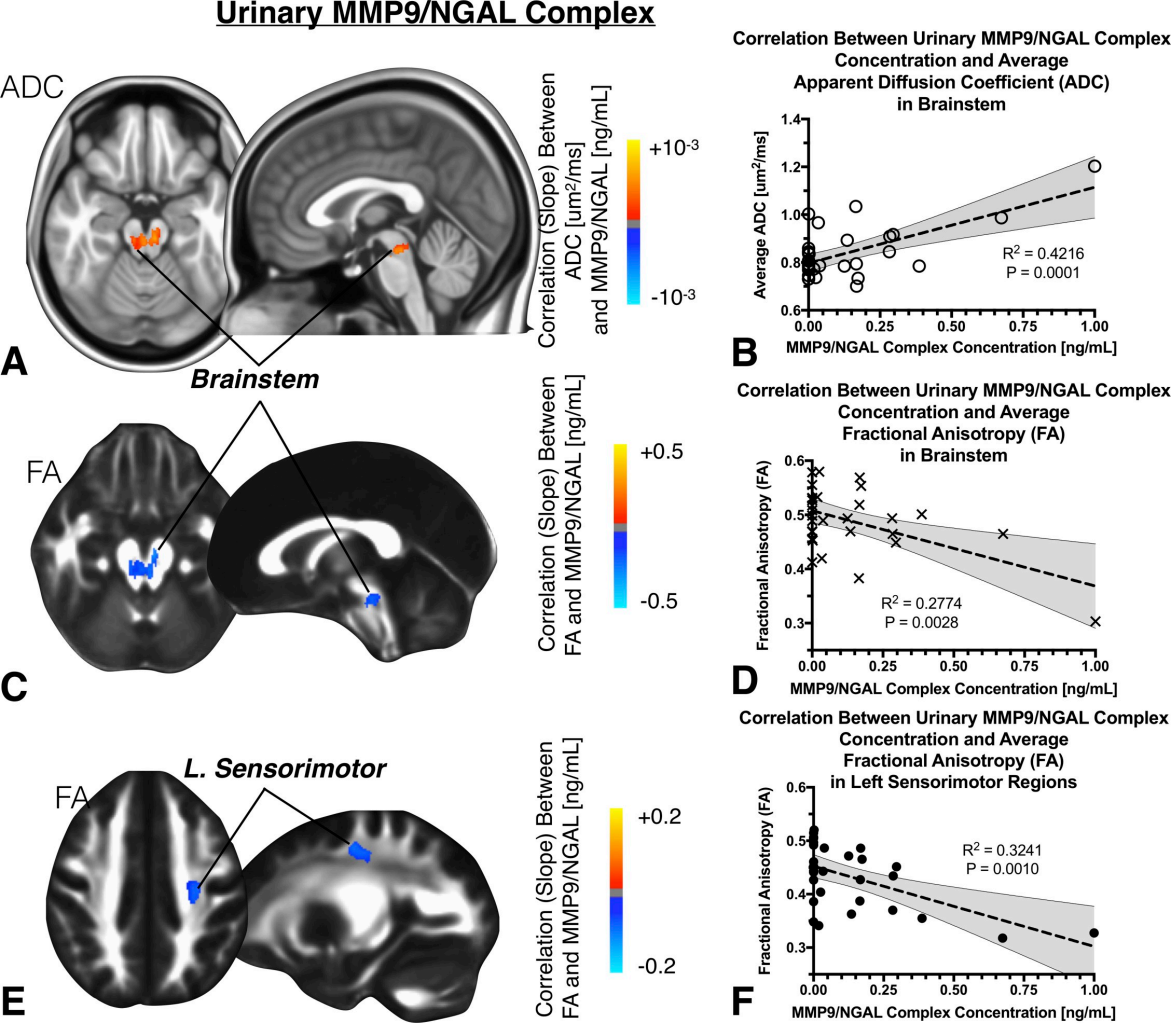
A) Anatomic localization of regions in the brain exhibiting a significant linear correlation between ADC and urinary concentration of the MMP9/NGAL complex, in similar brainstem regions as MMP9. B) Linear correlation between average ADC within this cluster and urinary MMP9/NGAL concentration. C) Anatomic localization of regions in the brain exhibiting a significant linear correlation between FA and MMP9/NGAL complex, similarly observed in the brainstem. D) Linear correlation between average FA within this cluster and urinary MMP9/NGAL concentration. E) Anatomic localization of regions in the brain exhibiting a significant linear correlation between FA and MMP9/NGAL complex in the left sensorimotor region. F) Linear correlation between average FA within this cluster and urinary MMP9/NGAL concentration.

Comparable to regions associated with urinary MMP9 concentration, a 965uL brainstem cluster, containing the DRN and LCC, was found to have a strong correlation between ADC and urinary concentration of the MMP9/NGAL complex (**Fig 4A and 4B;** *R*^*2*^ *= 0*.*4216*, *P = 0*.*0001*). An 916uL cluster within a similar brainstem region was identified as having a significant association between FA and MMP9/NGAL complex (**Fig 4C and 4D;** *R*^*2*^ *= 0*.*2774*, *P = 0*.*0028*). In addition to these brainstem regions, a single cluster within the left sensorimotor region (704uL) also demonstrated a significant association between FA and MMP9/NGAL concentration (**Fig 4E and 4F;** *R*^*2*^ *= 0*.*3241*, *P = 0*.*0010*).

### Urinary concentration of VEGF and cerebral DTI measurements

**Fig 5:**
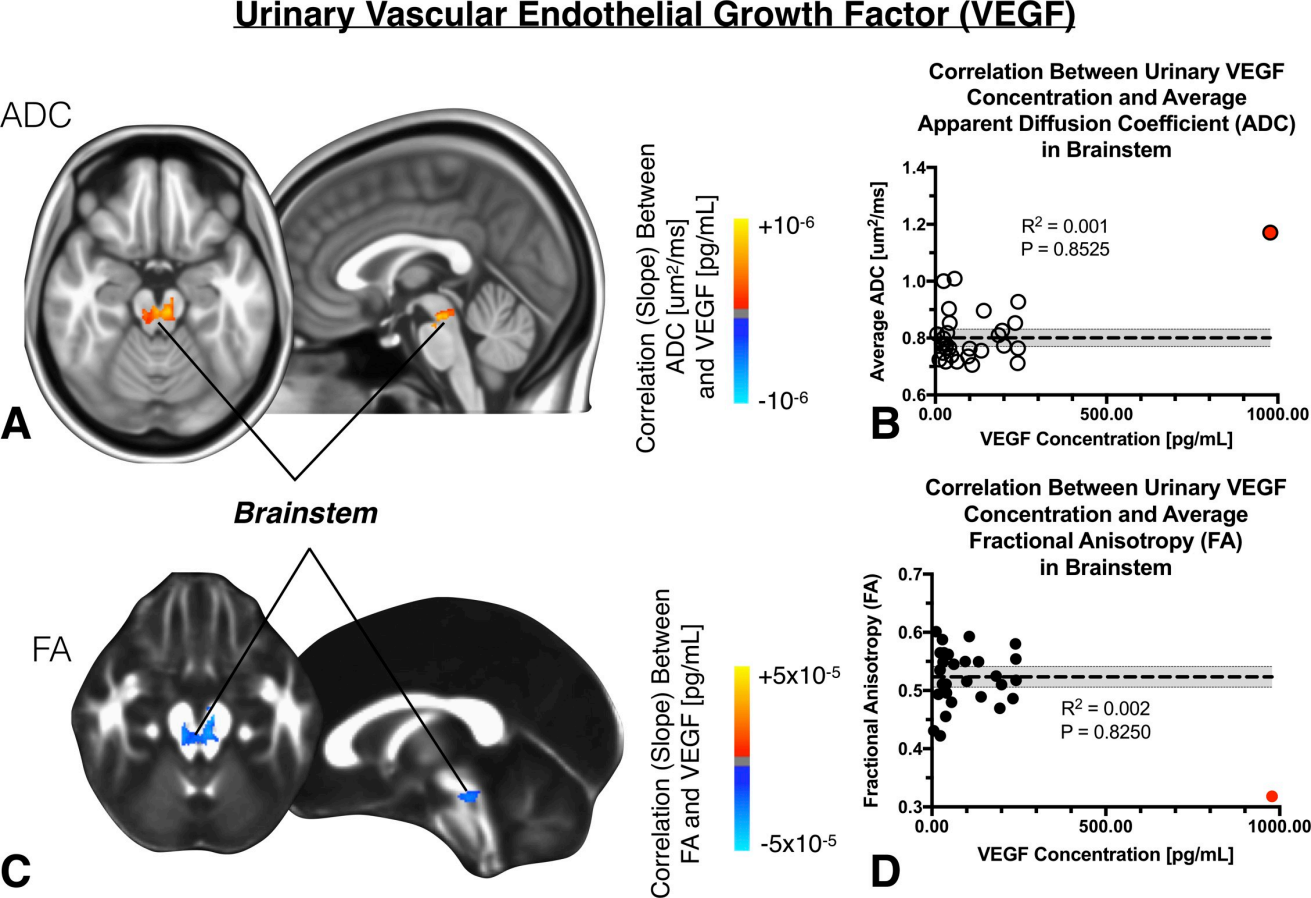
A) Anatomic localization of regions in the brain exhibiting a significant linear correlation between ADC and urinary concentration of vascular endothelial growth factor (VEGF) within the brainstem. B) No linear correlation between average ADC within this cluster and urinary VEGF concentration was observed after outlier correction. C) Anatomic localization of regions in the brain exhibiting a significant linear correlation between FA and VEGF. D) Similar to ADC, no linear correlation between average FA within this cluster and urinary VEGF concentration was observed after outlier correction. (Outliers = red circle).

Lastly, we examined the association between urinary VEGF concentration and DTI measurements within the brain. Initial results suggested a strong positive correlation between ADC and VEGF concentration localized to the midbrain (**Fig 5A;** *volume = 981uL*), but this trend was driven by a single outlier patient (red circle), as once this outlier was removed there was no apparent relationship (**Fig 5B;** *R*^*2*^ *= 0*.*001*, *P = 0*.*8525*). Equally, we observed an initial association between FA and VEGF concentration within the brainstem (**Fig 5C;** *volume = 992uL*) that was found to be driven by a single outlier patient (**Fig 5D;** *R*^*2*^ *= 0*.*002*, *P = 0*.*8250*).

## Discussion

**Fig 6:**
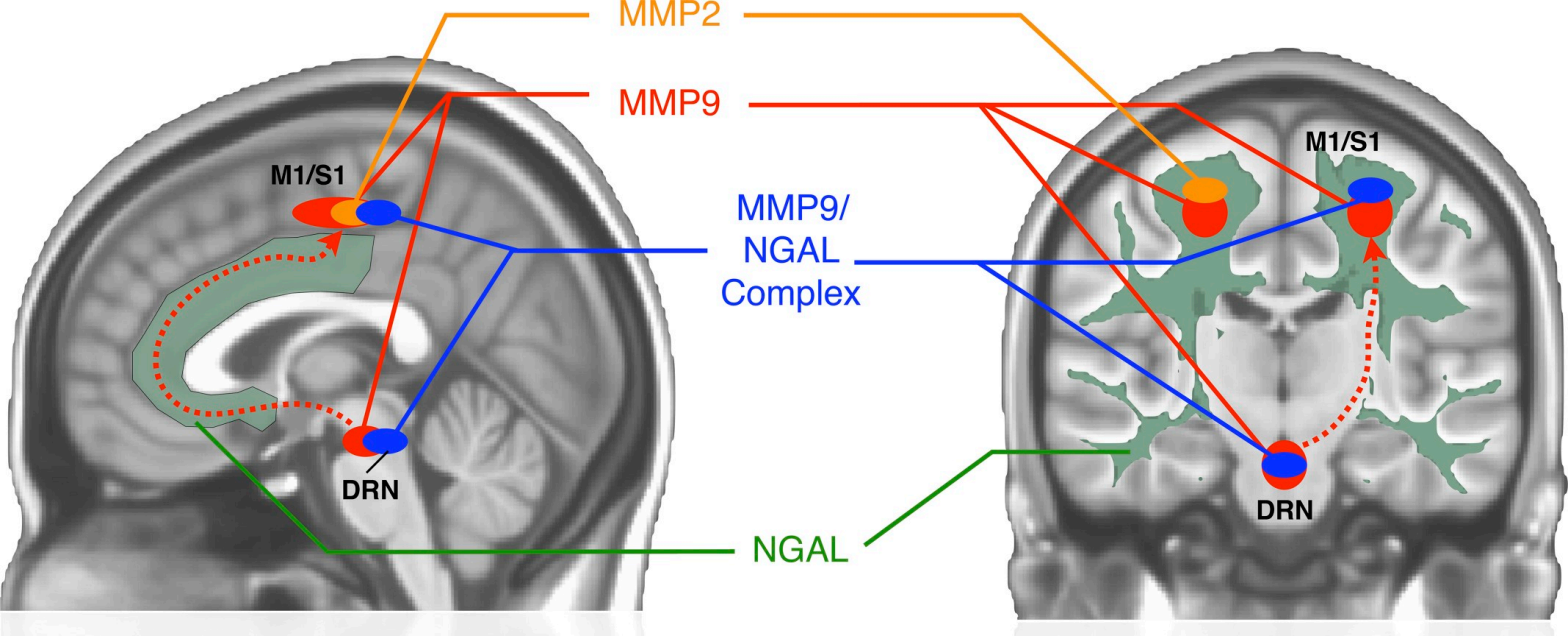
Illustration of observed associations between urinary protein levels and DTI brain measures. In UCPPS patients with elevated MMP9 as a result of local or systemic inflammation, excitotoxicity and eventual death of neurons in the dorsal Raphe nuclei (DRN) may occur, as MMP9 is known to lead to excitotoxicity in glutamatergic neurons and 2/3 of neurons in the DRN are both glutamatergic and serotonergic. The DRN is known to be the primary serotonergic center for the brain and projects throughout the brain including sensorimotor (M1/S1) regions. Increased concentration of MMP9, NGAL, MMP9/NGAL complex, and altered serotonin all modulate aspects of brain plasticity through manipulation of dendritic projections, altering long-term potentiation (LTP), and other synaptic changes. Additionally, altered serotonin levels, MMP9, and NGAL have all independently been linked to other conditions including anxiety and depression, which are also commonly observed in patients with UCPPS.

The current study identified distinct regions of the brain that appear to be microstructurally altered in proportion to the concentration of urinary biomarkers associated with inflammation, tissue remodeling and/or the vasculature (**Fig 6**). In particular, we found changes in diffusion MR measurements most consistent with degenerative processes, namely an increase in ADC and decrease in FA, in the brainstem and bilateral sensorimotor white matter regions with increasing metabolite concentrations. Additionally, widespread alterations in ADC and FA were associated with changes in NGAL, suggesting elevated NGAL may be associated with a high degree of axonal or neural plasticity. While the goal of the present study was not to determine the mechanism(s) by which brain structure and these specific urinary biomarkers might interact, we provide a number of potential possibilities, outlined below, that may help to explain the underlying interaction and may provide promising paths for further research.

The degenerative changes within the brainstem associated with increasing MMP9 and MMP9/NGAL concentration in the urine that were observed in this current study appear to be localized to the DRN, which is the largest serotonergic nucleus and provides substantial serotonin innervation throughout the brain. Alterations in serotonin levels have been observed in chronic pain [37], and decreased serotonin activity [38, 39] and smaller physical size of the DRN have both been observed in depression [40, 41], a common comorbidity observed in patients with chronic pain [42–44], including those with UCPPS [45]. Further, the use of selective serotonin reuptake inhibitors (SSRIs) have shown some efficacy in treating both chronic pain and depressive symptoms [46], including UCPPS [47], suggesting a potential link between DRN degeneration, serotonergic alterations, and UCPPS.

Serotonergic projections from the DRN extend to numerous regions within the cerebral cortex, including somatosensory and motor regions [48]. There is sufficient evidence to suggest cortical serotonin levels influence synaptic and dendritic plasticity [49, 50], including those associated with anxiety and depression [51–53]. This synaptic and dendritic plasticity is also facilitated by MMP9 [54–58], which is also elevated in the serum of patients with depression [59]. In the brain, it is widely appreciated that MMPs activate neuroinflammatory pathways both directly and indirectly, contribute to neuroinflammation-mediated neurotoxicity and compromise vascular integrity [60]. For example, elevated MMP9 is known to cause excitotoxicity through glutamate dysfunction [61], and while the DRN is commonly associated with serotonin activity, approximately 2/3 of serotonergic neurons in the DRN also release glutamate [62, 63]. It is therefore conceivable that some patients with UCPPS may have elevated MMP9 associated with an inflammatory process, which in turn may both contribute to the neurodegenerative changes to the DRN directly through glutamatergic excitotoxicity as well as facilitate synaptic plasticity near sensorimotor cortical regions to compensate for chronic afferent pain input. The consequential damage to serotonergic neurons in the DRN may then induce a reduction of cortical serotonin levels, resulting in additional comorbidities, including anxiety and depression, commonly observed in UCPPS.

Microstructural changes in the brain corresponding to changes in urinary NGAL concentration were notably more diffuse and widespread when compared with the spatially-specific changes in the brainstem and somatosensory regions associated with MMP9 and MMP9/NGAL complex. Although less is known about its effects in the brain, NGAL, or lipocalin-2, is an inflammatory signaling molecule with a diversity of functions in the brain [64]. Consistent with our observations of widespread microstructural alterations proportional to NGAL levels, other studies have shown that NGAL tightly controls dendritic spine formation and maturation [65]. It is also important to note that we have previously reported that NGAL binds to MMP9 in the form of the MMP9/NGAL complex and in doing so, protects MMP9 from autodegradation [66] thereby protecting its biological activities. Additionally, NGAL has been linked to anxiety, depression, pain hypersensitivity, emotional instability, psychological stress, cognitive function, and locomotive behavior [64, 67, 68], many of which are common issues for patients with UCPPS [45].

Another possible explanation is that changes in brain networks that are related to bladder control create conditions in the bladder that alter the expression of the urinary biomarkers described here. Multiple neuroimaging studies performed by the MAPP network suggest that primary differences between UCPPS patients and heathy controls appear in sensorimotor, viscerosensory, and brainstem regions related to the control of bladder function [10, 12, 13, 69, 70]. These changes may be related to a general upregulation of pelvic floor muscle tone observed in UCPPS patients [71–76]. It is possible that upregulated pelvic floor activity could create mechanical conditions similar to bladder outlet obstruction. Partial urinary outlet obstruction is known to generate changes in gene expression in DRG neurons [77]. For example, exposing detrusor smooth muscle cells to increased pressure creates a time-dependent decrease in MMP-9 activity [78]. It is therefore possible that the changes in brainstem structure in areas related to control of pelvic floor muscle and bladder function may change the mechanical properties of the bladder environment, leading to changes in urinary biomarker expression. Taken together, these results suggest that a bidirectional loop may exist between brainstem regions associated with micturition and the bladder. Additionally, results suggest that the urinary proteins analyzed in this report have the potential to complement neuroimaging approaches in the study of brain function in UCPPS patients.

Another potential mechanism to explain the observations in the current study may be degenerative changes in the brain resulting from neuroinflammation, particularly due to glial activation, which was recently targeted for imaging in chronic lower back pain patients using positron emission tomography (PET) [79]. Several studies using DTI have found colocalization of neuroinflammatory processes with FA and ADC alterations. For example, several studies have found colocalization between DTI measures and microglial activation in the brain as assessed through PET using various radiolabels, including [^11^C]-PBR28 activity colocalizing with decreased FA in both amyotrophic lateral sclerosis [80] and in primary lateral sclerosis [81], and associations between global levels of [^11^C](R)-PK11195 radiolabel PET and DTI measures in multiple sclerosis [82] and stroke[83]. While these studies lend credence to the idea that DTI measures are altered in the presence of neuroinflammatory processes, nonetheless the relationship between the peripheral protein markers detected in urine used for this study and neuroinflammation may indeed be tenuous.

There are several limitations to this study, which limit the conclusions including the small sample size. Out of 259 urinary samples from UCPPS patients used in our previous study [15] and 45 patients with high quality DTI used in a previous neuroimaging study [12], only 30 of these patients had both urinary protein measurements and DTI data available for analysis in the current study. While not an aim of this study, another limitation is related to lack of causality in that it is unclear as to whether the urinary markers reflect local organ (bladder, prostate, or pelvic floor) dysregulation. Finally, the limited spatial resolution of DTI limits the identification of the exact anatomical substrate of the identified brainstem changes. For example the monoaminergic DRN and the LCC are closely adjacent, and have strong functional connections.

## Conclusions

In this study, we observed that specific urinary protein levels were highly correlated with brain diffusion MRI measurements of microstructural integrity. Results suggest elevated MMP9 or MMP9/NGAL in UCPPS may correlate with degeneration of neurons in brainstem nuclei, possibly through glutamatergic excitotoxicity, while also facilitating synaptic plasticity in the cerebral cortex. Both NGAL and MMP9 are known to modulate plasticity in the brain and damage to monoaminergic neurons may explain comorbidities including depression that are often associated with UCPPS. Future investigations exploring the potential association between urine protein biomarkers and brain changes as measured by other neuroimaging techniques are further warranted to validate and expand our findings, including structural and functional MRI analyses focusing on measurement of atrophic changes in the brainstem or motor cortex and their association to MMP2, MMP9 and MMP9/NGAL protein levels.
